# A trial platform to develop a tailored theory-based intervention to improve professional practice in the disclosure of a diagnosis of dementia: Study protocol [ISRCTN15871014]

**DOI:** 10.1186/1748-5908-1-7

**Published:** 2006-03-31

**Authors:** Martin P Eccles, Robbie Foy, Claire H Bamford, Julian C Hughes, Marie Johnston, Paula M Whitty, Nick Steen, Jeremy G Grimshaw

**Affiliations:** 1 Centre for Health Services Research, University of Newcastle upon Tyne, 21 Claremont Place Newcastle upon Tyne, UK; 2 Ottawa Health Research Institute, Ottawa Hospital, Ottawa, Canada; 3 Northumbria Healthcare NHS Trust, North Tyneside General Hospital, Rake Lane, North Shields, Tyne and Wear, UK; 4 School of Psychology, University of Aberdeen, William Guild Building, King's College Aberdeen, UK

## Abstract

**Background:**

For people with dementia, care should include an explanation of the diagnosis to individuals and their carers, and information about the likely prognosis and possible packages of care. However, this is neither routine nor inevitable, and there is wide variation in the practice of disclosure. The aim of this study is to develop a tailored theory-based intervention to promote appropriate disclosure of diagnosis of dementia.

**Methods:**

There are three objectives. Objective 1 is to define and develop an appropriate model of disclosure; this will be addressed using a multidisciplinary consensus development process. Objective 2 is to identify factors that influence disclosure of diagnosis; a questionnaire based upon theoretical constructs from a range of behavioural theories will be developed and members of old age mental health teams will be surveyed. The analysis will identify those factors that best predict intention to disclose a diagnosis to a person with dementia. Objective 3 is to develop and pilot test a theory-based intervention to promote disclosure of diagnosis that targets attitudes, beliefs and actions most amenable to change. Objective 3 will use the results of Objectives 1&2 to design and pilot test an intervention to improve the process of and increase the proportion of individuals receiving a diagnosis of dementia, for members of old age mental health teams. This work will lead to a proposal for a randomised controlled trial of the intervention.

## Background

Approximately 600,000 people in the UK have dementia. This is 5% of the population aged 65 and older, and 20% of those aged 80 and older [1]. By 2026 this figure is predicted to be 840,000. Dementia is associated with major social and economic costs, including those to families and carers. The UK National Service Framework (NSF) for Older People states that the improved care of people with dementia depends on early recognition and management [1]. Such care should involve a sensitive and accurate explanation of the diagnosis to individuals and carers, and information about the likely prognosis and possible packages of care.

Appropriate disclosure of a diagnosis to individuals with dementia is important for three reasons. First, from an ethical perspective, people with dementia, like other patients, have a right to know their diagnosis. At present, most carers are told the diagnosis [2], but this is not the case for people with dementia themselves [3]. Indeed, disclosure is less likely in dementia than in other terminal conditions, such as cancer. Earlier disclosure, supported by advocacy groups, allows the opportunity to plan family, financial, legal and long-term care arrangements. Second, many people with dementia want to know their diagnosis or receive more information about their illness [4-6]. Third, disclosure can facilitate decisions about treatment. Whilst this is increasingly important in the advent of therapies to slow disease progression, anecdotal evidence suggests that patients prescribed anti-dementia medication are not inevitably told their diagnosis. The UK Alzheimer's Society Consumer Network has identified issues around early diagnosis and care as research priorities.

It is a consistent finding that changing clinical practice is unpredictable and can be a slow and haphazard process. Over the last decade a considerable body of literature has been published suggesting that a range of interventions (e.g. reminder systems, interactive education) can be effective in changing health care professionals' behaviour [7]. However, studies have substantial heterogeneity of interventions used, targeted behaviours, and study settings that make generalising their findings to routine healthcare settings problematic. Moreover, there is no underlying generalisable taxonomy for either research or service settings by which to characterise individuals, settings and interventions. The assumption that clinical practice is a form of human behaviour and can be described in terms of general theories relating to human behaviour offers the basis for a taxonomy for Implementation Research. For example, the effectiveness of interventions may be influenced by factors such as health professionals' *beliefs *or *perceived control over their practice *– generalisable concepts that can be used across different contexts. Two steps are necessary to design a theory-based intervention for a behaviour change trial [8]. One is to identify modifiable factors underlying professional behaviour in order to identify those processes to target with an intervention (process modelling), and the second is to understand how interventions might work and be optimised (intervention modelling). These respectively correspond to the theoretical phase and the modelling and exploratory trial phases of the UK Medical Research Council (MRC) Framework for the development and evaluation of complex interventions [9,10].

### Work conducted to date

In the clinical area of dementia we have conducted a systematic review that indicates wide variation in the reported practice of disclosure of dementia among health professionals [3]. Four main factors appear to influence disclosure: (1) patient characteristics (e.g. age, ability to retain the diagnosis); (2) nature of the dementia (e.g. severity, diagnostic uncertainty, availability of disease-slowing therapies); (3) structural factors (e.g. time); and (4) clinician factors (e.g. perceived value of disclosure) [11-19]. We have conducted a detailed primary and secondary care case note review to look for symptoms that would allow the earlier diagnosis of dementia. We also have conducted focus groups and in-depth interviews with a range of health professionals, carers, and people with dementia [20]. These suggest potential ways of improving current practice and highlight the importance of the process of referral and testing in preparing people with dementia and their carers for a diagnosis.

In the area of Implementation Research we have conducted several pragmatic RCTs of behaviour change strategies and have conducted both process and intervention modelling studies. Although these methods are familiar to psychology, their use with healthcare professionals and their integration into implementation trials is novel. We will use these methods to develop an optimised, theory based intervention – targeting modifiable factors – to increase diagnostic disclosure of dementia. This work will lead to a randomised controlled trial of the intervention.

### Aim

To develop a tailored theory-based intervention to promote appropriate disclosure of diagnosis of dementia.

### Objectives

(1) To define and develop an appropriate model of disclosure for dementia; (2) To identify, within a theoretical framework, factors that influence disclosure of a diagnosis of dementia by members of old age mental health teams (MHTs); (3) To develop a theory-based intervention that promotes appropriate disclosure by targeting those factors identified in (2) that are amenable to change.

## Methods

### Objective 1: Defining an appropriate model of disclosure for dementia

Disclosure is ideally a process tailored to individuals' receptiveness and needs for information. A group of ten relevant stake-holders (i.e., psychiatrists, community psychiatric nurses, patient group representatives, general practitioners) will be convened and will use a structured consensus method [20] to define an appropriate model of disclosure, including consideration of both positive and negative effects. This will be informed by: available documents (e.g. NSF for Older People [1]), findings from our qualitative work [18], further interviews with people with dementia and two carer focus groups, and a request for examples of good practice in the Alzheimer's Society national newsletter.

### Objective 2: To identify, within a theoretical framework, factors that influence old age mental health teams' disclosure of a diagnosis of dementia

#### Design

Postal questionnaire survey.

#### Study sample

Disclosing a diagnosis of dementia is predominantly the responsibility of a consultant old age psychiatrist, although a range of other team members contribute to the process. We will identify involvement in disclosure within the postal questionnaire survey (below) of old age MHT members. This survey will also permit assessment of the feasibility of identifying and surveying MHT members. There are about 420 old age MHTs in the UK, and they will have differing structures and working patterns. For Objective 2, we will sample the 60 MHTs in the North of England and Yorkshire, and another 60 randomly selected from the rest of the UK. The former will be subsequently approached for recruitment to the RCT; the latter will provide data about the generalisability of the planned trial participants.

### Theory selection

The theories (Theory of Planned Behaviour (TPB), Social Cognitive Theory (SCT) and Implementation Intentions (II)) have been chosen for three reasons. First, they have all been rigorously evaluated in other settings. Second, they all explain behaviour in terms of factors amenable to change (e.g., beliefs, attitudes, and perceived external constraints). Third, they all include non-volitional components that assume individuals do not always have complete control over their actions. According to TPB, the strength of a behavioural intention is determined by attitudes towards the behaviour (in this case disclosure), subjective norms based on the perceived views of other individuals or groups (i.e. perceived social pressure); and perceived behavioural control, encompassing beliefs about self-efficacy (the ability to perform an action) and wider environmental factors that facilitate or inhibit performance [21]. SCT considers self-efficacy and individuals' goals in explaining behaviour [22]. Self-efficacy is highly predictive of a wide range of behaviours and can be enhanced by experience of success, observation of others' performance, or persuasive communications. II suggests that motivated individuals with a clear action plan are more likely to act. IIs are both predictive and a means of changing behaviour [23].

### Questionnaire design

We will develop questions that explore constructs within the theories. Table 1 provides illustrative examples of variables, measures and items for each.

**Table 1:** Illustrative examples of theories, variables, measures and items

**Theory**	**Predictor variable(s)**	**Measures**	**Illustrative items**
Theory of Planned Behaviour	Attitude towards disclosure; subjective norms perceived behavioural control; intention	Items developed from qualitative work [18] using standard question formats	Attitudes – outcome beliefs: *Being given a diagnosis of dementia will be beneficial to the patient*.
Social-Cognitive Theory	Self-efficacy about disclosure; goals relevant to disclosure	Items developed from qualitative work [18] using standard question formats	Self-efficacy: *I am confident in my ability to disclose a diagnosis of dementia sensitively*.
Implementation Intentions	Action plans for disclosure	Open questions with simple coding for presence of action plans	*Have you thought about increasing disclosure of dementia diagnosis? How will you go about this?*

Questions for TPB and SCT will be assessed using multi-item scales, with items rated on seven-point Likert scales. The *content *of questions about control beliefs (those that influence the ability of professionals to disclose a diagnosis) relating to TPB, self-efficacy and goal beliefs relating to SCT will be drawn from existing qualitative data. Evidence of II will be ascertained using open questions about how the respondent normally discloses, which will be coded for evidence of "action plans" to disclose.

#### Outcomes

We will use two measures of outcome: behavioural intention and behavioural simulation. We will measure behavioural intention using standard methods, i.e. rating scales of likelihood, frequency or agreement with statements or questions about intention. Six clinical scenarios that vary combinations of relevant items of patients and diagnosis will be used to measure behaviour simulation.

#### Administration

Following piloting, the questionnaire will be distributed with reminders at two and four weeks. Surveys of UK mental health professionals with an interest in older age in the last decade have achieved acceptable response rates of between 73% and 88% [14,19,24-26]. Based on previous experience with theory-based questionnaires such as this, we anticipate the response rates will be lower. Therefore, we will offer a financial incentive of GB£20 for each returned completed questionnaire. MHT members will be asked to complete questionnaires independently (i.e. not together in teams). To conform with the Data Protection Act, we intend to identify and seek participation in the postal survey of professionals as follows: we shall contact the appropriate NHS Trusts and seek demographic profiles of local old age MHTs; we shall then send Trusts the appropriate numbers of introductory letters (not addressed to named individuals) and participant information sheets for distribution to members of local old age MHTs; when members of old age MHTs receive the letters, they can decide whether to opt in to the survey and return their names and preferred contact details to us in a pre-supplied stamped addressed envelope; we shall then send out survey questionnaires to those who have opted in. Under this plan, we should be able to identify our sample denominator (to allow calculation of response rates and sample representativeness), and send reminders and financial incentives to those who have opted in (so as to enhance our response rate).

#### Sample size and analysis

The analysis will allow us to explain variation in both team and individual level behaviour. The surveys will generate at least ordinal level quantitative data. The relationships between predictor (i.e. theoretical constructs and clinical discipline) and outcome measures (behavioural intention and simulation) will be assessed primarily using multiple regression analysis and structural equation modelling – a procedure that utilises the observed covariance matrix. In both cases, the analysis will take into account the hierarchical structure of the data. Power calculations for multiple regression analysis depend on the number of cases per predictor variable. A minimum sample size of 50 + 8 m, where m is the number of predictor variables, is recommended for testing the multiple correlation, and 104 + m for testing individual predictors [27,28]. We have approximately 10 predictor variables, requiring a minimum sample size of 130 per survey to test the multiple correlation, or 114 to test individual predictors. We intend to approach approximately 420 individuals that make up approximately 120 mental health care teams. This allows for a (worst case) response rate of around 50% – and for the lack of independence of responses from individuals within a team.

### Objective 3: Development and pilot test of a theory-based intervention to promote disclosure of diagnosis by old age mental health teams (ISRCTN 15871014)

#### Design and study sample

We will use the products of Objective 2 to identify the most promising elements of a potential intervention and then evaluate them using a randomised controlled design with MHTs. We will use members of MHTs that have previously responded in Objective 2, and augment the sample to reach the required size by randomly sampling within those MHTs not previously approached in Objective 2.

#### Identifying potential interventions

Objective 2 will identify the factors that are (a) modifiable and (b) the best predictors of behavioural intention. However, there will be a range of factors with these characteristics, and we will need to choose those factors which, in addition, can be modified by an intervention that is feasible. Therefore, we need to choose the two or three "best bets" (dependent on the results of objective 2) and simulate delivering them within a trial and examine their effects [29-31]. In these modelling experiments, elements of an intervention are manipulated within a randomised controlled design in a manner that simulates a real situation as much as possible. Interim endpoints (stated behavioural intention and simulation) are measured rather than changes in professional behaviour or healthcare outcome. This novel approach offers experimental control and the opportunity to vary elements of an intervention in order to better understand intervening variables and the effect on different outcomes. Behavioural intention has been incorporated into virtually all models of health behaviour as the single best predictor of subsequent health behaviour. In a review of 10 meta-analyses Sheeran demonstrated a consistent relationship between behavioural intention and subsequent behaviour, with intention explaining 28% of the variance in behaviour [32]. Members of MHTs will receive (or will have received) an initial survey as in Objective 2. Responders to this will be randomly allocated by team to receive one of up to three simulated interventions or a no intervention control. They will be instructed to open the intervention materials and then to complete a further theory-based survey as in Objective 2. Thus, we will identify the method that is most effective at changing professionals' beliefs and intentions. The sample size for a four-armed trial, powered to detect a difference between any two arms, is based upon the following assumptions: MHT as the unit of analysis, the outcome variable in the form of a score for the team, 80% power, and a type 1 error rate of 2.5% (rather than 5% to allow for multiple comparisons). Furthermore, we are aiming to detect a relatively large effect size of 0.8 on the basis that the modelling experiment eliminates some of the sources of variability associated with a 'definitive trial' (e.g. patient characteristics), and any smaller modelling effect size is unlikely to translate into a worthwhile effect in the definitive trial. Hence, we require four groups of 30 teams (120 in total). We will survey 240 teams (excluding those from North of England and Yorkshire) to allow for a 50% response rate in order to achieve the required sample size.

#### Pilot of outcome measurement

We will undertake a small scale postal survey of carers and people with dementia to help develop and pilot the feasibility of collecting outcome measures using this method in a definitive trial.

### Partnerships

We will explore the scope for maximal 'buy in' by a range of potential stakeholders. Representatives of the Alzheimer's Society have been consulted over the development of this proposal, and we aim to involve the Alzheimer's Society directly in the development of the intervention. The Faculty of Old Age Psychiatrists of the Royal College of Psychiatrists has agreed to appoint a member to liaise with us in regard to the design and conduct of the study.

### Predicted outcomes and follow-on opportunities

We propose to evaluate the intervention developed within a cluster randomised controlled trial. We will seek the participation of MHTs in the North of England. This platform work represents the first stage in engaging MHTs and will provide necessary information about the feasibility and appropriateness of the trial. Although we propose to randomise MHTs, Objective 2 will help demonstrate whether targeting MHTs, as opposed to individual psychiatrists only, is appropriate and feasible. The trial outcomes will include the proportion of people with dementia (and carers) aware of their diagnosis, quality of information provided, use of medical treatments, and the economic consequences of the intervention.

### Ethical and other implications

The study has MREC approval. The University of Newcastle operates a *Good Practice in Research Code *to ensure highest standards of integrity in research.

## Competing interests

The author(s) declare that they have no competing interests.

## Authors' contributions

All authors contributed to the ideas and writing of this paper. They have all seen and approved the final draft.
